# Neuroprotective effect of dexmedetomidine in a murine model of traumatic brain injury

**DOI:** 10.1038/s41598-018-23003-3

**Published:** 2018-03-21

**Authors:** Jin Wu, Todd Vogel, Xiang Gao, Bin Lin, Charles Kulwin, Jinhui Chen

**Affiliations:** 1 Department of Orthopaedics, the Affiliated Southeast Hospital of Xiamen University, Zhangzhou, China; 2 Department of Neurosurgery, Indiana University, Indianapolis, IN USA; 3 Spinal Cord and Brain Injury Research Group, Stark Neuroscience Research Institute, Indianapolis, IN USA

## Abstract

No FDA approved pharmacological therapy is available that would reduce cell death following traumatic brain injury (TBI). Dexmedetomidine (Dex) is a highly selective agonist of alpha-2 adrenergic receptors and has demonstrated neuroprotective effects in hippocampal slice cultures undergoing direct impact. However, no one has tested whether Dex, in addition to its sedative action, has neuroprotective effects in an animal model of TBI. Thus, in the present study, we investigated the effects of Dex on an animal model of TBI. Mice received different doses of Dex (1, 10, or 100 µg/kg bodyweight, n = 10 each group) or saline as control at 1 hour and 12 hours following TBI. The mice treated with Dex lost less cortical tissue than the control mice. Further analysis found that Dex treatment reduced cell death in the cortex and the hippocampus measured by Fluoro-Jade B (FJB) staining, prevented axonal degeneration detected by immunostaining with antibody against β-amyloid precursor protein (β-APP), and protected synapses from elimination with synaptophysin staining. Taken together, in an *in vivo* murine model of TBI, Dex at the dose of 100 µg/kg not only prevented tissue lesion and cell death, but also reduced axonal injury and synaptic degeneration caused by TBI.

## Introduction

Traumatic brain injury (TBI) takes a devastating annual toll, resulting in 52,000 deaths and 2.0 to 2.5 million emergency room visits annually in the USA. As the leading cause of death and disability in people ages 1 to 44, TBI is especially morbid for an otherwise young and healthy population^1,2^. Survivors suffer not only physical dysfunction, but also neurobehavioral disabilities^3^, as well as increased susceptibility to the development of neurodegenerative diseases, such as Alzheimer’s disease and Parkinson’s disease^4,5^. Permanent functional deficits in victims limit their productivities at home or during work, thus bringing about a heavy socioeconomic burden to families and communities. Thus, it is critical to develop therapeutic approaches to reduce TBI-induced brain damage.

In addition to the primary traumatic injury, TBI has been shown to involve secondary injury mechanisms including microvascular ischemia, autonomic dysregulation, and neuronal excitotoxicity^6–8^. Although numerous investigations have explored developing neuroprotective reagents^9–14^, none of them have been successful in clinical trials to date. Because no FDA approved pharmacological therapy is currently available to reduce cell death following TBI, it is critical to evaluate existing, FDA-approved agents for unrecognized therapeutic benefit in this disease process. Currently, pharmacologic sedation is the first-line treatment for elevated intracranial pressure in severe TBI.

Dexmedetomidine (Dex) is a highly selective alpha-2 adrenergic receptor agonist currently approved for sedation during mechanical ventilation in adults^15^. Dex has been used in patients with TBI and has shown no risk of affecting brain oxygenation^16^ or causing hemodynamic physiological changes^17^. More importantly, Dex interests neurosurgeons and neurologists alike because it has demonstrated neuroprotective effects in animal models of ischemia/reperfusion^18^ and excitotoxicity^19^. Additionally, multiple studies have indicated the value of autonomic modulation in animal TBI models, demonstrating the effectiveness of alpha agonists^20^, beta blockers^21^, and vagal nerve stimulation^22^. An *in vitro* study showed neuroprotection of Dex in hippocampal slice cultures undergoing direct impact^23^. However, there has been a paucity of investigation into the neuroprotective effects of Dex in animal traumatic models. No one has tested whether Dex, in addition to its sedative action, has neuroprotective effects in an animal model following TBI. In the present study, we evaluated the effects of Dex in a murine model of TBI to determine if it offers neuroprotective benefits.

## Materials and Methods

### Animals

Male C57 BL/6 mice were used in experiments at an age of 8 weeks. All procedures were performed under protocols approved by Indiana University’s Animal Care and Use Committee (IACUC). All methods were performed in accordance with the guidelines approved by Indiana University Institutional Biosafety Committee.

### Controlled Cortical Impact Traumatic Brain Injury

Eight-week-old mice (n = 76) were subjected to moderate, controlled cortical impact injury (CCI), as we previously described^24,25^. The mouse CCI model uses an electromagnetic impactor (Impact One^TM^, Stereotactic Impactor for CCI, Leica Microsystem, Illinois, USA). For this study, the contact velocity was set at 3.0 m/sec and the deformation was set at 1.0 mm. These settings resulted in an injury of moderate severity. Following injury induction, the skin incision was sutured. Sham (non-injured) mice (n = 18) were subjected to craniotomy, but did not receive a CCI injury.

### Dex treatment

After surgery, the CCI-injured mice (n = 60) were randomized for placement into saline or Dex treatment groups. The mice then received intraperitoneal (i.p.) injections of saline (n = 15), Dex 1 µg/kg (n = 15), Dex 10 µg/kg (n = 15), or Dex 100 µg/kg (n = 15) twice (at 1 hour, then again at 12 hours) after surgery. Twelve hours after the final injection (24 hours post-injury), 10 animals in each group were sacrificed for assessment of cell death with Fluoro-Jade B (FJB) staining, as well as axonal injury and synaptic degeneration assessment with immunofluorescent staining. The remaining mice in each group (n = 5) were sacrificed at 1 week after injury to measure cortical tissue lesion volume with cresyl violet staining. For behavior testing, the CCI-injured mice were placed randomly in 2 groups (n = 8 for each group) and received an i.p. injection of saline or Dex at a dose of 100 µg/kg of body weight. Sham surgery mice (n = 8) were used as a control.

### Tissue Processing

All animals were deeply anesthetized with Avertin and then perfused transcardially with cold saline, followed by a fixative containing 4% paraformaldehyde (PFA) in PBS. The brains were removed, post-fixed overnight in PFA, and cryoprotected for 48 hours in 30% sucrose. Serial 30 μm thick coronal sections were cut using a cryostat (Leica CM 1950), and stored at −20 °C. The sections were then processed for histopathological analysis.

### Histological Analysis

Fluoro-Jade B (FJB) staining was used for evaluating neuronal cell death and as immunofluorescent staining for examining the pathological changes. Cresyl violet staining was used for measuring cortical cavity volume as we previously reported^14,26–30^.

### Fluoro-Jade B Staining

Frozen sections were mounted on Superfrost plus slides (Fisher Scientific), were rinsed in distilled water for 2 minutes to rehydrate, and were transferred to a solution of 0.06% potassium permanganate for 20 minutes. The sections were then rinsed in distilled water for 2 minutes and placed in a 0.0004% FJB solution containing 0.1% 4′,6-Diamidino-2-phenylindole (DAPI, Sigma) for 20 minutes. Finally, the stained slides were washed in distilled water, dehydrated thoroughly, and then mounted with DPX (Sigma).

### Neuronal Cell Death Counting in the Cortex

The density of FJB-positive cells in the spared cortex surrounding the impact area following TBI was determined through a stereological analysis (10 mice for each group). Three sections per mouse in each group were chosen at 24 hours after TBI based on their close proximity to the epicenter of the impact, and the density of FJB-positive cells was quantified. Though rare, regions of tissue that were already dead and no longer had any positive FJB signals were not included in the measuring. Using Stereo Investigator software (MicroBrightfield Inc., Williston, VT), the tissue boundaries were defined and traced at 5× magnification on an Axio Imager M2 microscope (Zeiss). FJB-positive neuron counting was performed using a systematic sampling site method of the selected tissue area at 40× magnification for accurate recognition. The size for each site was set at 300 × 300 µm^2^ and the counting frame was 100 × 100 µm^2^. FJB-positive cells were selected based on morphology and fluorescent signal strength. After both the population of FJB-positive cells and the specified tissue volume (in cubic millimeters) were obtained, the density of cells per cubic micrometer was calculated using the following formula: cell density = (cell population)/(tissue volume #/mm^3^).

### Neuronal Cell Death Counting in the Hippocampus

Following the method used in our previous report^24^, 24 hours after TBI, the total number of FJB-positive cells in the dentate gyrus (DG) of the hippocampus was determined through a blinded quantitative histological analysis (10 mice for each group). Whole sets of 1 in 6 sections covering the entire extent of the hippocampal formation were selected for assessment with FJB staining. Every FJB-positive cell was counted and was determined under a fluorescent microscope with a 20× objective. Finally, the total number of FJB-positive cells on any given section were summed.

### Immunofluorescent Staining

Immunofluorescent staining was carried out according to our previous reports^27,28^. In brief, sections were rinsed in PBS 3 times (5 minutes each) and incubated in blocking buffer (0.1% Triton X-100, 1% bovine serum albumin, and 5% normal serum in PBS) for 1 hour at room temperature. Then, primary antibodies were added to blocking buffer and incubated with sections overnight at 4 °C. After 3 washes with PBS (5 minutes each), sections were incubated with the secondary antibody for 1 hour at room temperature and then treated with 4′,6-Diamidino-2-phenylindole (DAPI) for 2 minutes. Finally, sections were washed 3 times with PBS (5 minutes each), dried, and mounted using Fluoromount G (Southern Biotech, Birmingham, AL, USA). Primary antibodies and their final concentrations were as follows: β-APP antibody (1:1000, rabbit, Invitrogen, 36–6900), synaptophysin antibody (1:1000, mouse, Millipore, MAB5258), Iba1 antibody (1:200, goat, Abcam, ab5076), GFAP antibody (1:1000, mouse, Sigma, G3893), and NG2 antibody (1:200, rabbit, Millipore, AB5320). Secondary antibodies from Jackson ImmunoResearch Laboratories Inc. were all applied at the same dilution of 1:1000.

### Cresyl Violet Staining

Briefly, frozen sections were mounted on Superfrost plus slides (Fisher Scientific, Pittsburgh, PA, USA) and stained using 0.1% cresyl violet solution for 20 minutes. Stained sections were rinsed in double-distilled water quickly and were immersed in 95% ethanol for 2 minutes. Then, the sections were dehydrated in 100% ethanol and were cleared in 100% xylene twice (5 minutes each time). Finally, the sections were mounted with DPX for imaging.

### Cortical Cavity Volume Measurement

Briefly, whole sets of 1-in-6 series of 30-μm-thick brain sections (7 days after TBI, 180 μm apart) were stained with cresyl violet to show the spare cortex. Then, boundary contours of the contralateral and ipsilateral spare cortex were drawn using AxioVision software (version 4.8, Zeiss, Thornwood, NY, USA). The enclosed volume within the contours was measured, and the percent cortex of the cavity was calculated with the following formula: percentage of the cortical cavity = (contralateral cortex volume −  ipsilateral spare cortex volume)/(contralateral cortex volume) × 100%.

### Imaging

The images were taken at a primary magnification of 10×, 40×, and 63× using an invert microscopy system (Zeiss, Axiovert 200 M) interfaced with a digital camera (Zeiss, Axio Cam MRc5) controlled by a computer.

### Rotarod Test

For motor function analysis, the rotarod test was used. Twenty-four mice were tested, with 8 mice in each of 3 groups (Sham, TBI + saline, and TBI + Dex). During the test, each animal was allowed to remain stationary for 10 seconds at 0 rpm. The speed was steadily increased by 3 rpm in 10-second intervals until the maximum of 30 rpm was reached. There were 5 trials each day for each mouse with a 20-minute interval between trials. The first rotarod test was performed before surgery to obtain a baseline. After surgery, the rotarod test was administered 2 times, at 3 days and 7 days after surgery. Duration on the stage was recorded and analyzed.

### Statistical Analysis

All data are presented as mean ± standard error of the mean (SEM). The SPSS18.0 program was used for data analysis. For cell death and tissue cavity analyses, statistical significance was determined by one-way ANOVA followed by an LSD *post hoc* test. For behavior tests, statistical significance was first determined by repeated measures, followed by one-way ANOVA and an LSD *post hoc* test to analyze specific time points between the 3 groups.

Statistical significance was determined at the P < 0.05 level.

## Results

### Post-injury Treatment with Dex Significantly Decreased TBI-Induced Cortical Lesion

**Figure 1:**
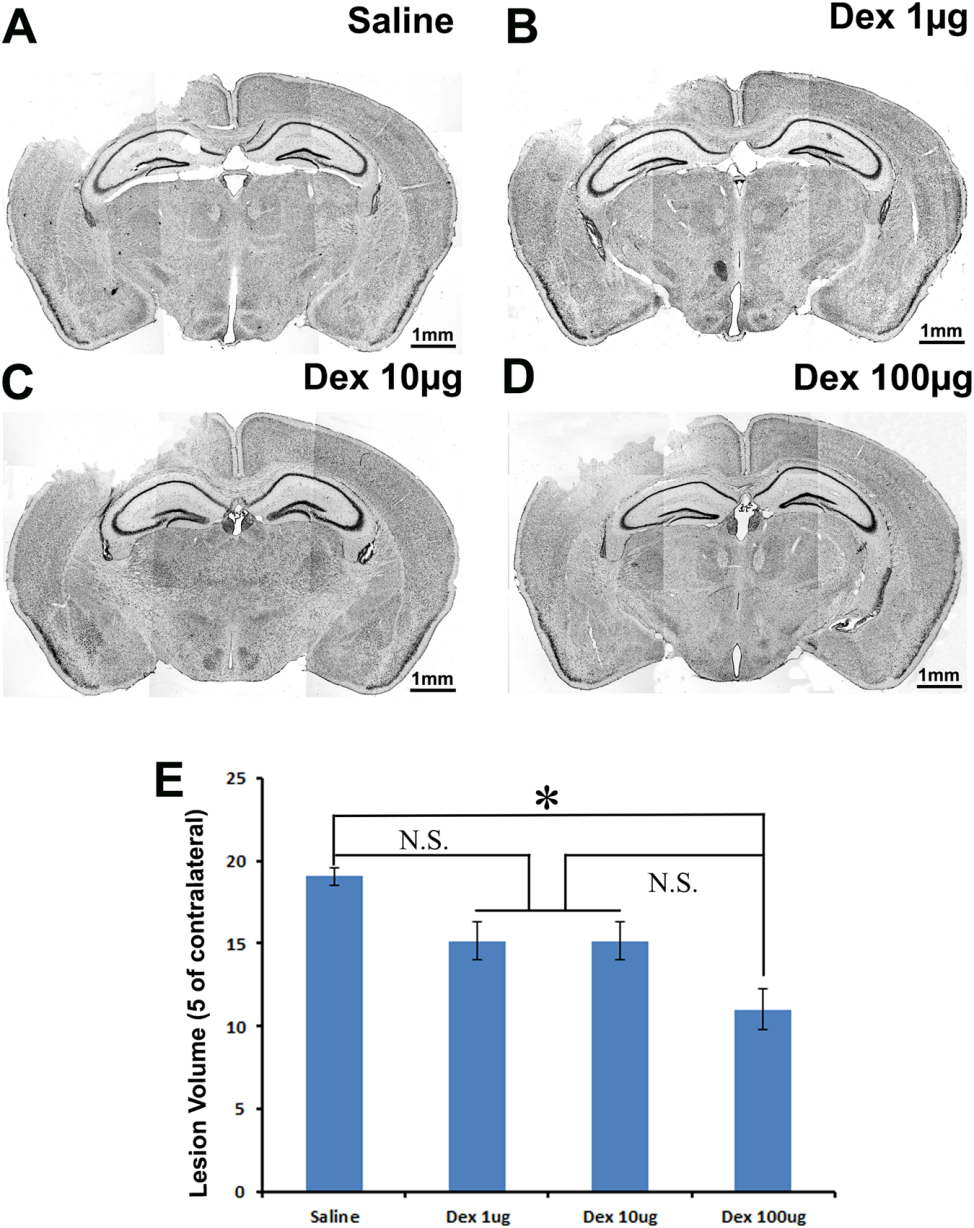
TBI-induced cortical cavity is reduced by intraperitoneal injection of 100 µg/kg Dex. Cresyl violet staining was performed to assess the cortical cavity in the brain that received saline or different concentrations of dexmedetomidine (Dex) treatment following moderate traumatic brain injury (TBI**)**. The representative images of (**A**) Cortical cavity of saline treatment mice after TBI. (**B**) Cortical cavity of 1 µg/kg Dex treatment mice after TBI. (**C**) Cortical cavity of 10 µg/kg Dex treatment mice after TBI. (**D**) Cortical cavity of 100 µg/kg Dex treatment mice after TBI. (**E**) Quantitative measurement of the cavity volume proportion of the damaged brain region. (n = 5, *p < 0.05).

The high incidence of neurological deficits and mortality after TBI has been attributed in large part to dramatic secondary cell death in the cortex that resulted in massive tissue loss in the cortex^31^. To examine the effects of Dex on tissue loss (cavity) in the cortex following TBI, the mice received an intraperitoneal (i.p.) injection with either saline or different concentrations of Dex (1 µg/kg, 10 µg/kg, or 100 µg/kg) at 1 hour and 12 hours after surgery. Seven days after surgery, the brains were removed to assess cortical cavity with cresyl violet staining. Based on the spared tissue volume, the cortex cavity volume was calculated to directly reflect tissue injury and protection. The results showed that the mice treated with saline lost 19.0 ± 0.5% of ipsilateral cortex tissue (Fig. 1A), while mice treated with Dex lost 15.1 ± 1.1% (1 µg/kg, Fig. 1B), 15.2 ± 1.1% (10 µg/kg, Fig. 1C), and 11.0 ± 1.2% (100 µg/kg, Fig. 1D) of ipsilateral cortex tissue, respectively. The cortex cavity volumes in TBI-injured mice receiving 1 µg/kg or 10 µg/kg Dex were less than TBI-injured mice receiving saline control. However, the differences did not reach statistical significance. The cortex tissue cavity volume was significantly reduced in TBI-injured mice receiving 100 µg/kg of Dex treatment compared to the saline treated control mice (Fig. 1E, P < 0.05). Two injections (i.p.) of Dex with 100 µg/kg dose following TBI decreased cortex cavity by 42.1% ((19.0–11.0%)/19.0%). These data indicated that Dex treatment showed a significant protective effect in a rodent model of TBI.

### Dex Treatment Prevents Neuronal Cell Death in the Cortex after Moderate TBI

**Figure 2:**
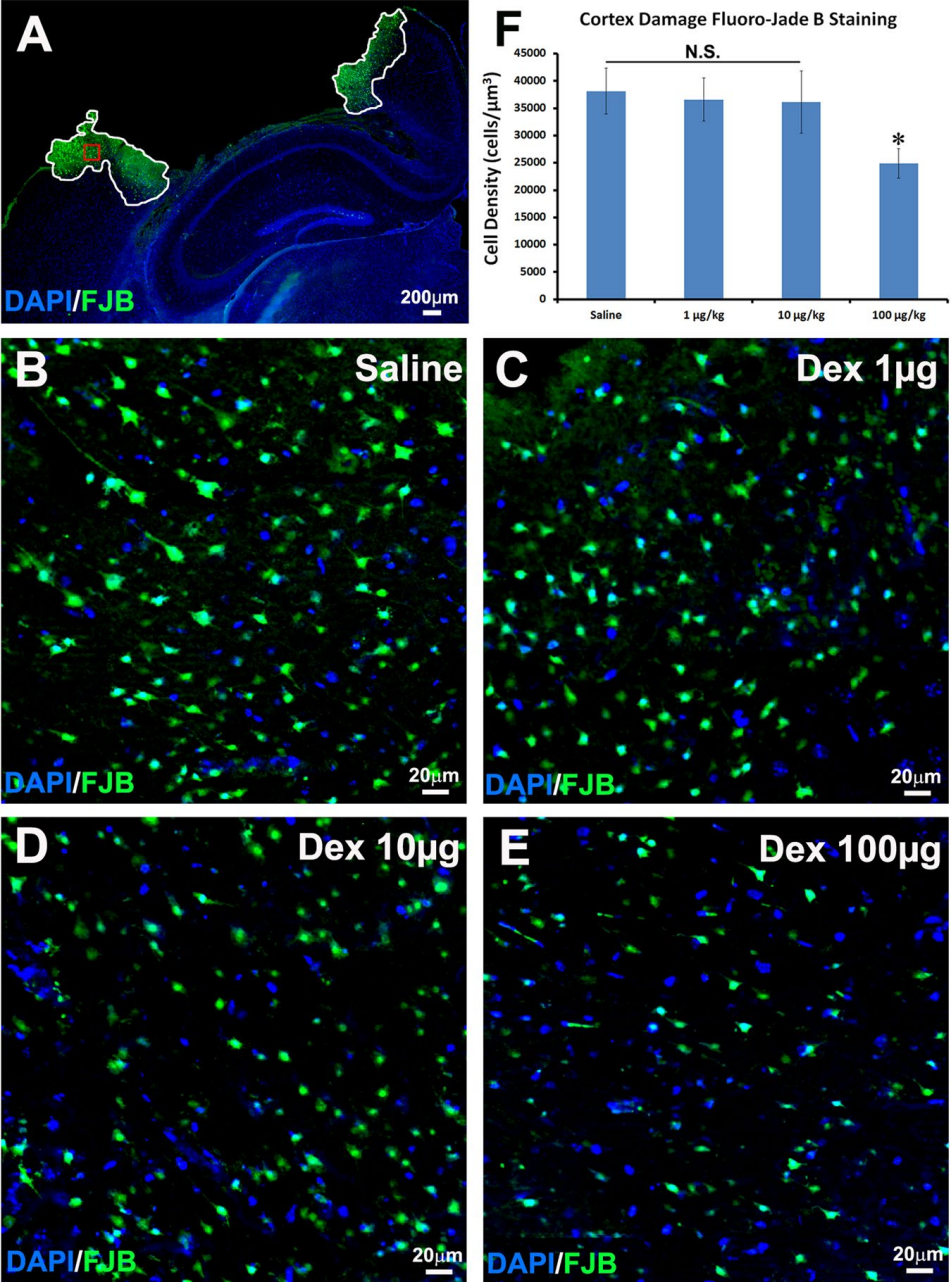
TBI-induced neuronal cell death in the cortex is decreased by intraperitoneal injection of 100 µg/kg Dex. Fluoro-Jade B (FJB) staining was performed to assess the neuronal cell death in the cortex that received saline or different concentrations of dexmedetomidine (Dex) treatment following moderate traumatic brain injury (TBI). (**A**) Schematic diagram of neuronal cell death counting. The regions enclosed by white lines were counting regions and the region enclosed by a red line was the region where higher magnification images were taken. (**B**) Neuronal cell death of saline treatment mice after TBI. (**C**) Neuronal cell death of 1 µg/kg Dex treatment mice after TBI. (**D**) Neuronal cell death of 10 µg/kg Dex treatment mice after TBI. (**E**) Neuronal cell death of 100 µg/kg Dex treatment mice after TBI. (**F**) Quantitative measurement of the neuronal cell death in the cortex. (n = 10, *p < 0.05).

We further assessed the cell death in the spared cortex by FJB staining; a schematic diagram of neuronal cell death counting is shown in Fig. 2A. The results showed that a significant difference was found in the density of FJB-positive cells in the cortical area around the lesion site between the saline control (38130.8 ± 4212.6/µm^3^, Fig. 2B and F) and the 100 µg/kg Dex treatment group (24877.9 ± 2678.8/µm^3^, Fig. 2E and F). There were no statistical differences between the 1 µg/kg Dex treatment (36577.2 ± 3946.3/µm^3^, Fig. 2C and F), 10 µg/kg Dex treatment (36157.7 ± 5704.0/µm^3^, Fig. 2D and F), and saline control groups. These results suggested that 100 µg/kg Dex dosing protected neurons in the cortex from death triggered by moderate TBI.

### Dex Treatment Prevents Neuronal Cell Death in the Hippocampal Dentate Gyrus after Moderate TBI

**Figure 3:**
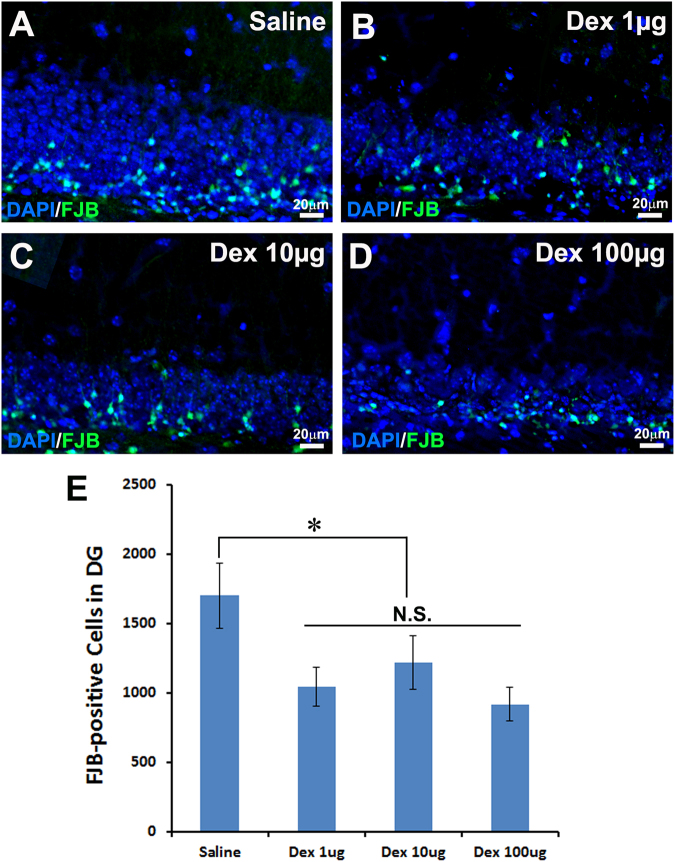
TBI-induced neuronal cell death in the hippocampus is reduced by intraperitoneal injection of 100 µg/kg Dex. Fluoro-Jade B (FJB) staining was performed to assess the neuronal cell death in the hippocampus that received saline or different concentrations of dexmedetomidine (Dex) treatment following traumatic brain injury (TBI). (**A**) Neuronal cell death of saline treatment mice after TBI. (**B**) Neuronal cell death of 1 µg/kg Dex treatment mice after TBI. (**C**) Neuronal cell death of 10 µg/kg Dex treatment mice after TBI. (**D**) Neuronal cell death of 100 µg/kg Dex treatment mice after TBI. (**E**) Quantitative measurement of the neuronal cell death in the hippocampus. (n = 10, *p < 0.05).

TBI not only causes cell death in the cortex, but also induces significant cell death in the hippocampus. To quantify cell death in the hippocampus, FJB staining was performed. The results showed that the FJB-positive cells in the hippocampus decreased as the dose of Dex increased. The number of FJB-positive cells in the DG of hippocampus was 1701.1 ± 235.5 (saline group, Fig. 3A), 1214.8 ± 193.0 (1 µg/kg Dex group, Fig. 3B), 1047.0 ± 140.2 (10 µg/kg Dex group, Fig. 3C), and 917.2 ± 119.6 (100 µg/kg Dex group, Fig. 3D), respectively. Unlike what we observed in the cortex, we found that Dex showed significant protection against hippocampal granular neuron cell death even at the low dose of 1 µg/kg. These data suggested that Dex treatment protected the hippocampus from injury at a much lower dose than in the cortex.

### Dex Treatment Prevents Axonal Damage in the Cortex after Moderate TBI

**Figure 4:**
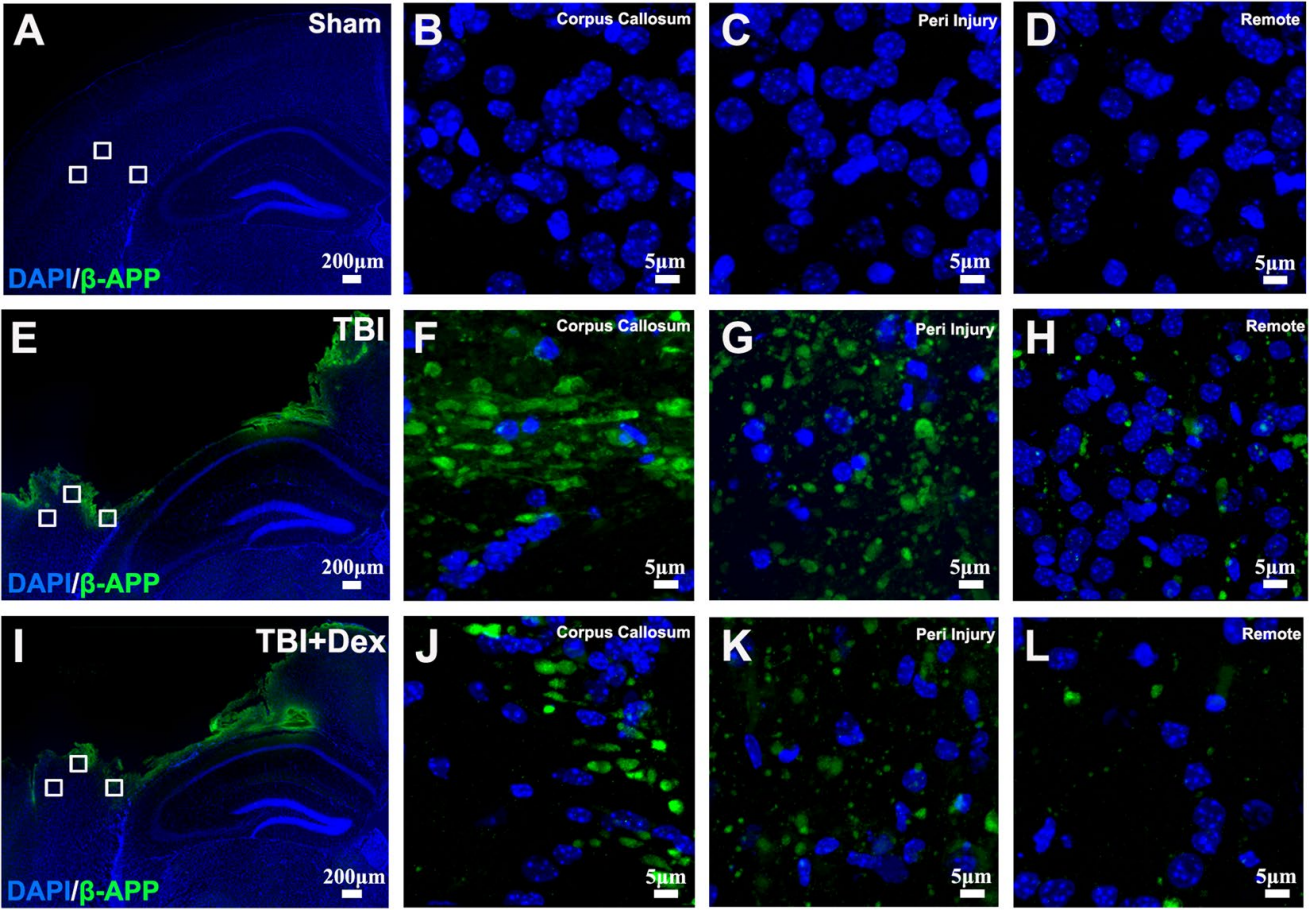
Post-injury Dex treatment prevents axonal damage in the cortex. Representative images showed the β-APP labeled damaged axon in the corpus callosum, peri-injury, and remote area of cortex of sham-injured, traumatic brain injury (TBI), and dexmedetomidine (Dex)-treated conditions. (**A–D**) Sham control mice showed no β-APP staining of cortex. (**E–H**) After TBI, significant axonal damage is detected with β-APP staining. (**J–L**) Dex treatment reduced the intensity of β-APP staining.

Axonal injury is often observed following TBI. Therefore, we assessed the potential of Dex to protect against secondary axonal damage. The results above indicated that Dex at the dose of 100 µg/kg not only decreased cortical lesion, but also prevented neuronal cell death in the cortex and in the hippocampus induced by moderate TBI. Thus, we determined whether Dex at this dose could protect axons from injury. After receiving moderate TBI, the mice had two 100 µg/kg Dex treatments at 1 and 12 hours after injury. The mice were then sacrificed at 24 hours after injury for assessing axonal injury with β-APP staining, which is a sensitive method to detect early axonal injury^32^. In the sham group, no β-APP immunoreactivity was observed in the cortex (Fig. 4A), including the corpus callosum (Fig. 4B), peri-injury region (Fig. 4C), and remote region (400 µm away from epicenter) (Fig. 4D). Twenty-four hours after moderate TBI, strong immunoreactive β-APP-positive axonal profiles appeared in the cortex (Fig. 4E) indicating dramatic axonal damage after moderate TBI. The corpus callosum area revealed β-APP immunoreactivity in the form of thick and short filaments (Fig. 4F) and the peri-injury (Fig. 4G) and remote regions (Fig. 4H) of the cortex revealed β-APP immunoreactivity in the form of small globules and granules (Fig. 4G). Treatment with Dex significantly reduced β-APP staining (Fig. 4I–L). As shown in Fig. 4I, the Dex treatment group exhibited weaker β-APP immunoreactivity in the cortex after moderate TBI. The immunoreactivity was in the form of fewer and smaller globules and granules in the corpus callosum (Fig. 4J) and the peri-injury region (Fig. 4K), while the remote region was almost negative for β-APP staining (Fig. 4L). We did not detect positive β-APP signals in the contralateral hemisphere of TBI brain either with saline or Dex treatment 24 hours after TBI (Suppl. Figure 1a–c). These results demonstrated that 100 µg/kg Dex treatment could prevent axonal damage in the cortex after moderate TBI.

### Dex Treatment Prevents Synaptic Degeneration in the Injury Area

**Figure 5:**
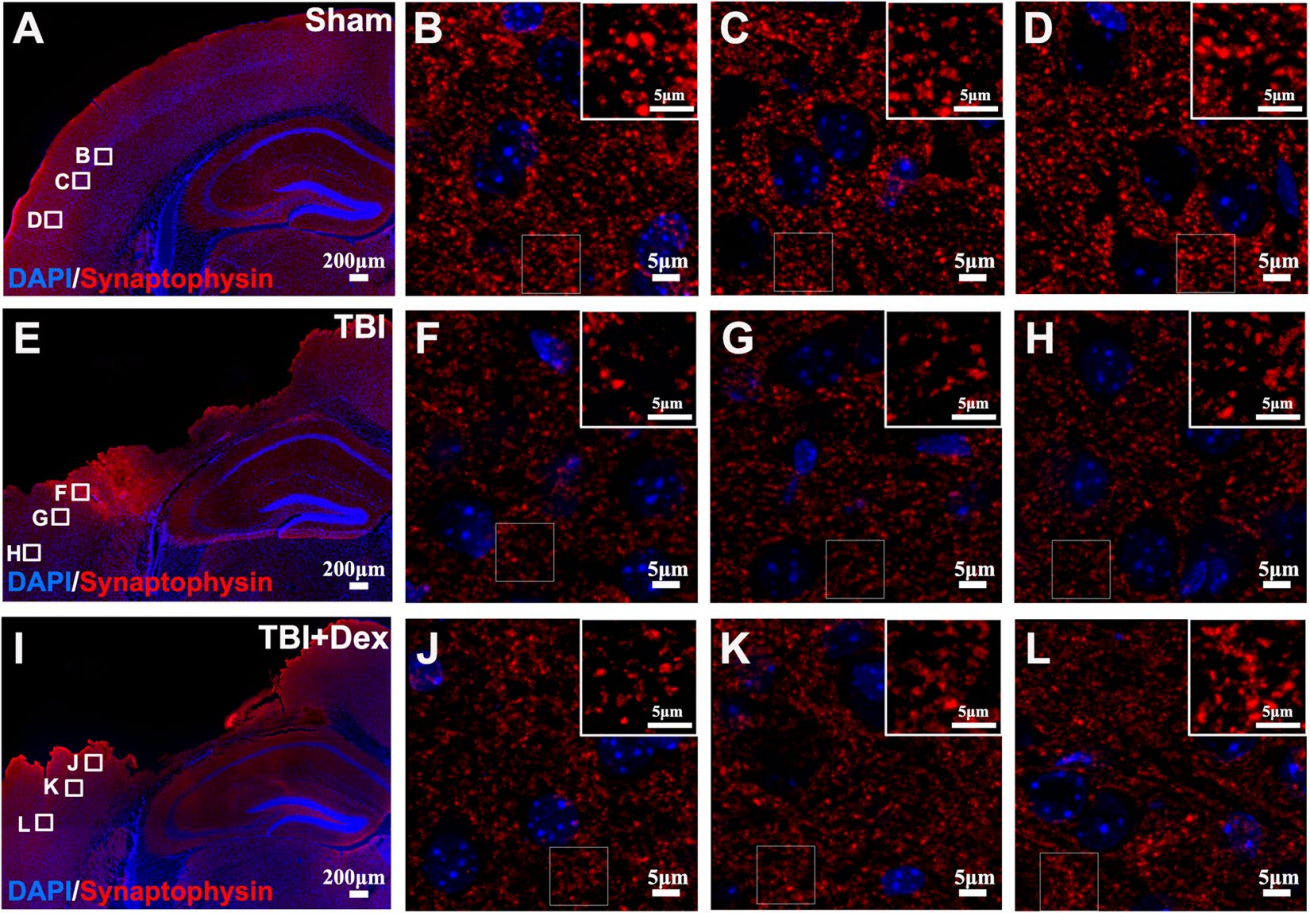
Dex treatment protected synaptic degeneration in the cortex after moderate TBI. Analysis of intensity of synaptophysin staining of the selected tissue area was performed at 10× magnification first, and then 3 regions in the cortex of each group were chosen for accurate recognition at 60× magnification according to the distance from the lesion cortex. (**A**) Synaptophysin staining in the sham group at 10× magnification. (**B–D**) Synaptophysin staining in the sham group at 60× magnification. (**E**) Synaptophysin staining in the traumatic brain injury (TBI) group at 10× magnification. (**F**) Synaptophysin staining in the TBI group 200 µm from the lesion cortex. (**G**) Synaptophysin staining in the TBI group 400 µm from the lesion cortex. (**H**) Synaptophysin staining in the TBI group 800 µm from the lesion cortex. (**I**) Synaptophysin staining in the 100 µg/kg dexmedetomidine (Dex) treatment group at 10× magnification. (**J**) Synaptophysin staining in the 100 µg/kg Dex treatment group 200 µm from the lesion cortex. (**K**) Synaptophysin staining in the 100 µg/kg Dex treatment group 400 µm from the lesion cortex. (**L**) Synaptophysin staining in the 100 µg/kg Dex treatment group 800 µm from the lesion cortex.

To further determine whether Dex has the preventive effects on synaptic degeneration, immunostaining with an anti-synaptophysin antibody was performed to detect presynaptic boutons. Three regions in the cortex were chosen for evaluating synaptophysin staining according to the distance from the edge of the cortex cavity (200, 400, and 800 µm) (Fig. 5A,E, and I). In the sham control animals, synaptophysin immunostaining was observed in a punctate distribution, consistent with localization of presynaptic boutons (Fig. 5B–D). Cell nuclei showed negligible synaptophysin immunoreactivity (Fig. 5B–D). In the TBI-injured brains (Fig. 5E–H), the intensities of synaptophysin staining and numbers of synaptophysin-positive puncta were reduced at the corresponding anatomic positions in the cortex, indicating that moderate TBI results in synaptic degeneration in the injury area. Dex treatment partially rescued synaptic degeneration indicated by increasing intensity of synaptophysin staining (Fig. 5I–L). When we further assessed the intensity and density of synaptophysin-positive signals in the contralateral side of the cortex, we found no detectable changes after TBI or after Dex treatments (Suppl. Figure 1d–f). The results indicated that Dex treatment prevented synaptic degeneration in the injury area.

### Dex Treatment Improved Behavior Outcomes

The above data showed that Dex treatment reduced the cortex cavity, prevented cell death both in cortex and hippocampus, and mitigated axonal and synaptic degeneration. These neuroprotective effects on cellular and tissue levels may contribute to neurobehavioral improvement. Therefore, we then further evaluated motor function using the rotarod test.

**Figure 6:**
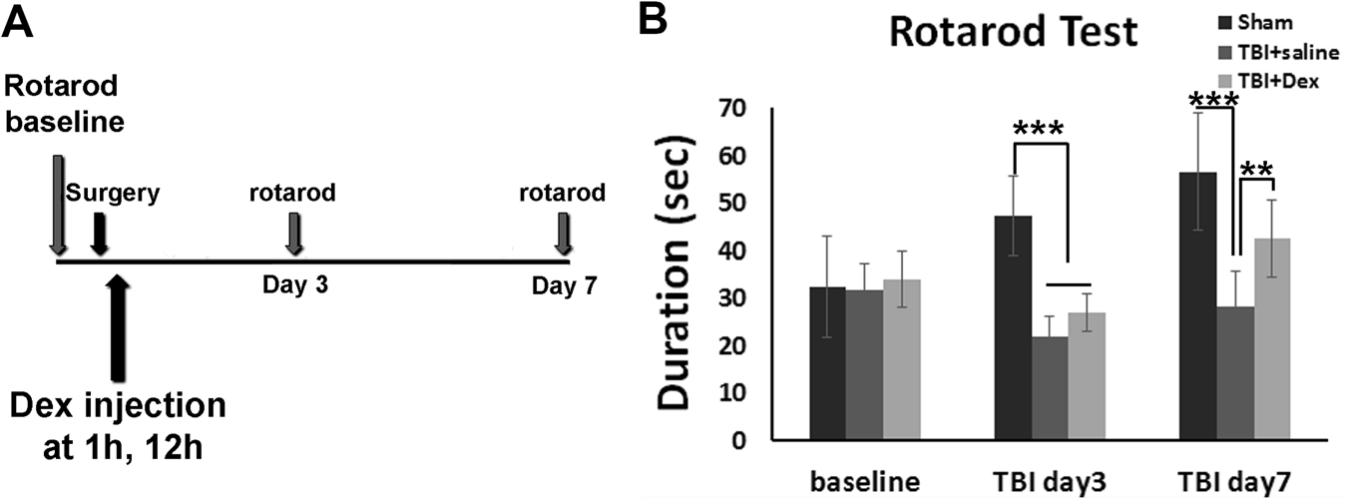
Dex treatment improved motor function outcomes after moderate TBI. (**A**) Schematic diagram of the time line of Dex treatments and motor function evaluation using the rotarod test. (**B**) Rotarod test was given to mice of 3 groups (sham, TBI + saline, and TBI + Dex) at 3 and 7 days after surgery. On the third day after surgery, the duration of both injury groups decreased significantly compared to the sham group, while the Dex treatment group showed the attenuated decline of duration compared to the saline treatment group. Seven days after injury, the duration of the Dex treatment group significantly increased and was much higher than the saline treatment group (n = 8 for each group, **p < 0.01, ***p < 0.001).

Before surgery, we assessed the motor performance of the mice onthe rotarod test to determine a baseline. Their duration on the stage was recorded and analyzed. Data showed that their durations on the stage were similar between different groups before surgery (Sham 32.27 ± 4.77 seconds; TBI + saline 31.74 ± 2.08 seconds; and TBI + Dex 33.83 ± 2.23 seconds). After surgery, the rotarod test was given 2 times at 3 days and 7 days, respectively (Fig. 6A). On the third day after surgery, the duration of the Sham group was increased compared to baseline (from 32.27 ± 10.66 seconds to 47.23 ± 3.77 seconds); this increased duration may have been due to the mice acquiring skill. Meanwhile, the duration of the TBI group with saline treatment showed a dramatic decrease (from 31.74 ± 2.08 seconds to 21.78 ± 1.71 seconds, p < 0.001). Dex treatment resulted in some degree of attenuation of duration dropping after TBI at this time point compared to the saline group, but did not reach statistical significance (26.91 ± 1.52 seconds compared to saline treatment 21.78 ± 1.71 seconds, p > 0.05). Seven days after TBI, duration in all groups increased, because of spontaneous recovery. The Dex treatment exhibited a much faster recovery rate than saline control. The duration of the Dex treatment group increased to 42.6 ± 3.09 seconds at this time point, which was significantly higher than the saline treatment group (saline 28.29 ± 2.77, p < 0.01, Fig. 6B). This result demonstrated that Dex treatment improved the motor function recovery following moderate TBI.

## Discussion

TBI is the major cause of death in young adults and often leads to substantial cognitive impairment, motor dysfunction, and epilepsy. Besides the severity of primary damage, secondary damage is an important determinant of a patient’s outcome. The secondary damage includes an entire cascade of cellular, chemical, tissue, and blood vessel changes in the brain that contribute to further destruction of brain tissue^33^. Numerous studies have expanded our knowledge of TBI’s pathophysiological events and could potentially serve as the basis to refine established strategies or even define new treatments. However, there is no effective treatment for the disorders caused by TBI.

Currently, pharmacologic sedation is the first-line treatment for severe TBI to control intracranial pressure. An ideal agent for sedation in the TBI patient population would be fast-acting with a short elimination half-time, allow for serial clinical examinations, accomplish adequate sedation in a dose-dependent fashion, be neuroprotective, have anti-epileptic properties and analgesic properties, and have no adverse effects on major organ systems. However, common agents include propofol and benzodiazepines, which interfere with serial neurologic evaluations, cause significant respiratory depression and hypotension, and may prolong time spent intubated in Intensive Care Units (ICU)^34^.

Unlike propofol^35^ and the benzodiazepines^36^, which act through the GABA_A_ receptor, dexmedetomidine (Dex) is an alpha-2 receptor adrenergic agonist with high selectivity about 7 to 8 times that of clonidine. Moreover, it is an FDA approved drug with a relatively short elimination half-time of almost 2 hours that is currently used in the sedation of humans. Furthermore, Dex is found to be safe and efficacious, and has no side effects on respiratory rate or oxygen saturations^37^. This enables it to be continued after extubation. However, there have been relatively few studies examining the role of Dex in patients with TBI. Aryan *et al*.^38^ reported its use in 39 neurosurgical patients in the ICU and concluded that Dex can be a safe and effective sedative agent for neurosurgical patients. Grof *et al*.^39^ undertook a small, prospective, observational study of patients receiving Dex in a neurosurgical ICU. Overall, the role of Dex in patients with TBI has yet to be adequately explored and more investigation is merited.

One *in vitro* TBI model study showed the neuroprotective effect of Dex in hippocampal slice cultures undergoing direct impact^23^. Thus, our study was aimed at demonstrating histological evidence of neuroprotection while using Dex *in vivo*. This investigation could potentially pave the way to clinical trials investigating Dex as an adjunct sedation in the TBI population. High-resolution images and quantified data showed that post-injury treatment with 100 µg/kg Dex reduced cortical tissue cavity volume in the TBI group. Further experiments proved that injecting a dose of 100 µg/kg Dex could also reduce neuronal cell death in the cortex and hippocampus in the TBI group. To further investigate more favorable effects of Dex at the dose of 100 µg/kg on moderate TBI, we focused on axonal damage, which is one of the most common and important pathologic features of TBI^40^. Our results indicated that 100 µg/kg Dex could prevent axonal damage dramatically in the cortex after moderate TBI. Our previous studies have shown moderate TBI causes acute synaptic degeneration in both the hippocampal dentate gyrus^41^ and cerebral cortex^42^. The findings have led us to wonder if Dex has preventive effects on synaptic degeneration. We found that Dex at the dose of 100 µg/kg did rescue synaptic degeneration in the cortex and improved functional recovery after moderate TBI. Taken together, Dex at 100 µg/kg dosing had a significant neuroprotective effect for this *in vivo* model.

A few study limitations are noted; the mechanism of how Dex exerts a neuroprotective effect on TBI remains unknown. Recent evidence suggests that this effect is mediated not only by the alpha-2 agonistic properties of Dex, but also by its binding to imidazoline I1-receptors^43^. In addition, obvious hypothermia was found in the mice that received a 100 µg/kg Dex injection. Thus, we speculate that the neuroprotective effects of Dex on TBI may not only be due to the activation of a signal transduction cascade linked to alpha-2 adrenergic receptors, but also due to the hypothermia induced by Dex. The mechanism of action of the demonstrated neuroprotective effect thus requires further investigation.

## Conclusion

Taken together, in an *in vivo* murine model of TBI, Dex at the dose of 100 µg/kg not only prevented tissue loss and cell death, but also reduced axonal injury and synaptic degeneration caused by TBI, resulting in improvement of motor function.

## Electronic supplementary material

dataset 1
