# Supplementary material for: Neuroprotective effect of dexmedetomidine in a murine model of traumatic brain injury
Source: Sci Rep. 2018 Mar 21;8:4935. doi: 10.1038/s41598-018-23003-3 (PMC5862953; doi:10.1038/s41598-018-23003-3)
Supplement: Supplementary file 1 — dataset 1 [file 41598_2018_23003_MOESM1_ESM.pdf]

Supplemental Data

Neuroprotective effect of dexmedetomidine in a murine model of traumatic brain injury

Jin Wu[1], Todd Vogel[2], Xiang Gao[3], Bin Lin[1], Charles Kulwin[2], Jinhui Chen[2,3*]

1 Department of Orthopaedics, the Affiliated Southeast Hospital of Xiamen University, Zhangzhou, China

[2] Department of Neurosurgery, Indiana University, Indianapolis, IN, USA

[3] Spinal Cord and Brain Injury Research Group, Stark Neuroscience Research Institute, Indianapolis, IN, USA

*Corresponding author:

Jinhui Chen, MD, PHD

Department of Neurosurgery

Indiana University

320 W. 15th Street, Indianapolis, IN 46202, USA.

Emails: chen204@iupui.edu

Supplemental figure 1

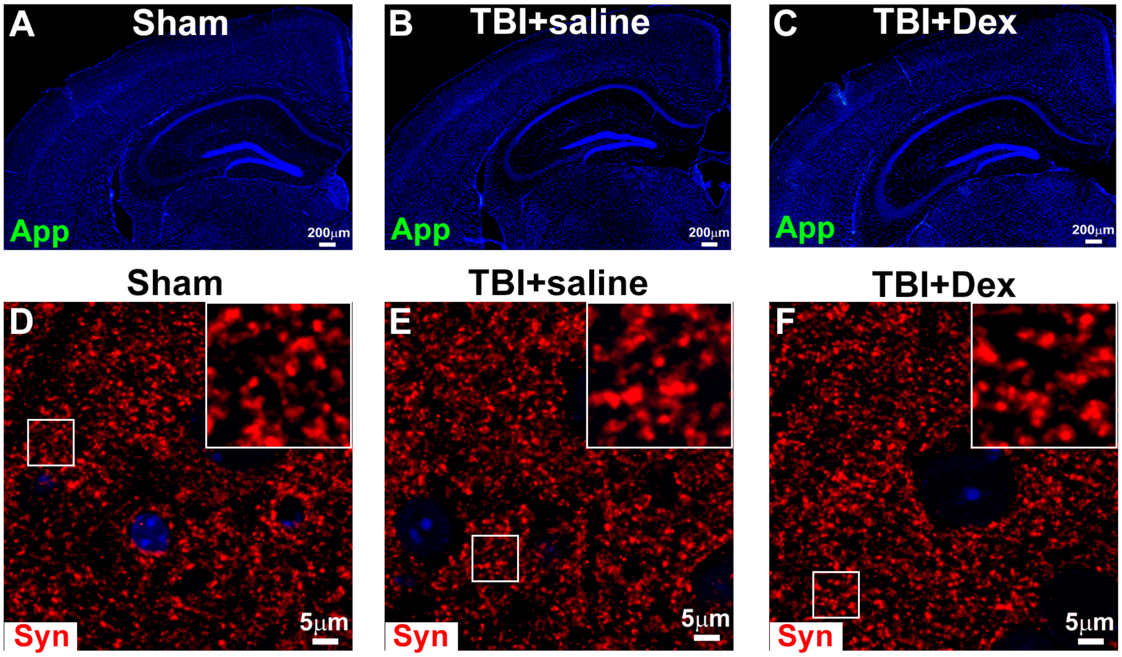

Supplement figure 1. β-APP and synaptophysin staining in the contralateral cortex 24hr after TBI.

Antibody against β-APP and synaptophysin were used to detect axonal degeneration and pre-synapses respectively. (A-C) There were no detectable β-APP-positive signals in the contralateral cortex among 3 groups. (D-F) There was no obvious change of synaptophysin signal in the contralateral cortex among 3 groups.
